# Transcriptome time-course analysis unravels the regulatory networks governing ratooning decline in sugarcane

**DOI:** 10.3389/fpls.2025.1739058

**Published:** 2026-01-12

**Authors:** Sisi Zhang, Feiyan Zhao, Zongtao Yang, Ting Yang, Yanye Li, Zhongfu Zhang, Jianming Wu, Jiayong Liu, Jun Deng, Yong Zhao, Yuebing Zhang

**Affiliations:** 1 National Key Laboratory of Tropical Crop Biology and Breeding, Kunming, Yunnan, China; 2 Sugarcane Research Institute, Yunnan Academy of Agricultural Sciences, Kaiyuan, Yunnan, China; 3 Fujian Agriculture and Forestry University, Fuzhou, Fujian, China; 4 Sugarcane Research Institute, Guangxi Academy of Agricultural Sciences, Nanning, Guangxi, China

**Keywords:** critical turning point, ratooning decline, sugarcane, time-course transcriptome, variety-specific response

## Abstract

**Introduction:**

Ratooning cultivation is the predominant production mode for sugarcane, yet ratooning decline represents a critical constraint limiting high and stable yields. To elucidate the molecular mechanisms underlying this phenomenon, this study aimed to reveal the critical period of sugarcane ratooning decline and variety-specific regulatory networks by integrating multi-year field yield data with transcriptome analysis.

**Methods:**

This study combined five consecutive years of field yield data (from plant cane to the fourth ratoon, PC–R4) with root time-course transcriptome data from three sugarcane varieties (YT93-159, YZ05-51, and YZ08-1609). Differentially expressed genes (DEGs) were identified, followed by Mfuzz time-course clustering, Weighted Gene Co-expression Network Analysis (WGCNA) to pinpoint key gene modules and hub genes, with validation by qRT-PCR.

**Results:**

The study revealed that the R2-R3 transition represents the critical turning point for ratooning decline. Compared to second ratoon (R2), yields of the three varieties decreased significantly by 14.3%, 12.64%, and 9.45% (*P* < 0.05), respectively, in R3 (third ratoon). Based on this critical period, comparative analysis between R3 and R2 identified 11,348, 20,638, and 21,977 differentially expressed genes (DEGs) associated with ratooning decline in the three varieties, respectively.Five highly overlapping gene modules (overlap rates: 48%–100%) were identified, yielding 25 hub genes, of which 15 exhibited peak expression during R3. These hub genes coordinately constitute a regulatory network encompassing energy metabolism, signal transduction, protein homeostasis, and defense responses. Notably, different varieties exhibited specific response pathways: YT93-159 specifically upregulated galactinol synthase (*GOLS*) to enhance osmotic adjustment; YZ05-51 primarily relied on thiosulfate sulfurtransferase (*STR*) to maintain cellular redox homeostasis; and YZ08-1609 upregulated ubiquitin ligase and proteasome subunit genes to strengthen protein quality control.

**Discussion:**

This study identifies the R2-R3 transition as the critical period of ratooning decline and uncovers variety-specific coping mechanisms at the molecular level. Different varieties exhibited distinct response pathways within the regulatory network, suggesting genotype-dependent adaptation strategies to ratooning stress. The key hub genes revealed here offer valuable molecular targets and genetic resources for breeding strong-ratooning sugarcane varieties, providing insights for improving sugarcane ratooning performance and sustainability.

## 1 Introduction

Sugarcane (*Saccharum* spp. hybrids) is one of the most important sugar and bioenergy crops worldwide, contributing more than 80% of global sugar production and accounting for approximately 90% of the raw material for sugar production in China (Healey et al., 2024; Li et al., 2024c). High and stable sugarcane yields are critical for ensuring sugar security. Ratoon cropping, as the primary cultivation mode in sugarcane production, can significantly reduce seedling and labor costs (Rossetto et al., 2022). Theoretically, the ratooning period can last for 5–8 years (Kandel et al., 2018; Yang et al., 2021). However, in the main sugarcane-producing regions of China, the phenomenon of “ratooning decline” is widespread (Ramburan et al., 2013), leading to the adoption of the cultivation pattern “one plant cane followed by two ratoon crops,” with ratooning duration rarely exceeding three years (Li and Yang, 2015). As ratooning continues, sugarcane typically exhibits symptoms including reduced sprouting rate, decreased effective stalk number, stunted plant growth, and lower single-stalk weight, resulting in progressive declines in both yield and quality that severely constrain the sustainable development of the sugarcane industry (Luo et al., 2025; Thomaz et al., 2022).

Ratooning decline is multifactorial process resulting from the interplay of agronomic, physiological, and molecular factors. At the agronomic level, key contributors include progressive soil nutrient depletion, deterioration of soil physicochemical properties, accumulation from soil-borne pathogens and pests, and suboptimal cultivation practices (Dlamini and Zhou, 2022; Xu et al., 2021). At the molecular and physiological level, roots, as the core organ for ratoon sprouting, exhibit a series of stress responses under continuous ratooning, including shifts in energy metabolism from growth-oriented to maintenance-oriented, hormonal signaling imbalances, activation of protein degradation pathways, trade-offs between defense responses and growth/development, and significant declines in nutrient uptake efficiency (Arif et al., 2019; Bartels et al., 2007; Feng et al., 2022; Hostetler et al., 2023; Jia et al., 2022; Karlova et al., 2021; Prinsi et al., 2018). These processes are intricately interwoven, collectively constituting the complex syndrome of ratooning decline.

However, these identified factors and mechanisms fail to adequately explain the temporal dynamics of ratooning decline. Field observation commonly show that yield decline is not a linear process but often exhibits an accelerating trend after a specific year, suggesting that ratooning decline may involve a critical transition phase from gradual to abrupt decline (Dlamini and Zhou, 2022; Silva et al., 2007). Nevertheless, the specific timing of this turning point, its underlying molecular mechanisms, and the variety-specific response mechanisms during this process remain unclear. Furthermore, the significant variation in ratooning ability among different sugarcane varieties implies a molecular regulatory basis reflecting genetic diversity (Abu-Ellail et al., 2019; Aitken et al., 2008; Wang et al., 2023). Resolving these issues is essential for understanding the mechanisms of ratooning decline and providing molecular targets for breeding improved ratooning varieties.

In recent years, transcriptomics has demonstrated significant potential in studying regeneration and decline in perennial crops. Transcriptome profiling of regenerating rice revealed the pivotal role of hormone signaling pathways in regulating regeneration capacity (Hu et al., 2025), while transcriptomic studies on continuously cropped alfalfa elucidated coordinated regulation between root metabolic networks and hormonal signals (Lu et al., 2020). These findings collectively suggest that regeneration or decline processes in perennial crops commonly involve core biological mechanisms including energy reallocation, signaling network reprogramming, and defense-growth trade-offs (Arif et al., 2019; Huot et al., 2014; Jia et al., 2022; Stitt and Zeeman, 2012). Despite these advances, sugarcane ratooning decline research remains constrained by fragmented transcriptomic studies that examine single varieties at individual times points, lacking systematic temporal profiling across multiple genotypes and consecutive ratoon cycles (Thirugnanasambandam et al., 2017; Xu et al., 2021; Zhao et al., 2025). Consequently, three critical knowledge gaps persist: the timing of molecular tipping points during decline progression remains undefined; the regulatory networks coordinating multiple processes orchestrating these transitions remain uncharacterized; and genotype-specific adaptive strategies conferring differential ratooning resilience remain unexplored.

To address these knowledge gaps, we conducted a five-year field experiment integrating yield data from plant cane (PC) to fourth ratoon (R4) with time-course root transcriptome profiling across three sugarcane varieties exhibiting contrasting ratooning capacities (YT93-159, YZ05-51, and YZ08-1609). Our study aimed to: (1) identify the critical temporal window when ratooning decline accelerates; (2) delineate the molecular signatures of root transcriptome reprogramming and the coordinately disrupted regulatory networks underlying this transition; and (3) dissect genotype-specific molecular strategies conferring differential ratooning performance. This integrative temporal-molecular approach not only establishes a mechanistic framework for understanding ratooning decline biology but also nominates candidate hub genes and targeted intervention strategies for precision breeding of ratooning-resilient sugarcane cultivars capable of sustaining productivity across extended ratoon cycles.

## 2 Materials and methods

### 2.1 Experimental materials, design, and sample collection

A five-year field positioning experiment was conducted at the main scientific research base of the Sugarcane Research Institute, Yunnan Academy of Agricultural Sciences (Kaiyuan City, Yunnan Province, China (23.70°N, 103.25°E; elevation 1,051 m). Three sugarcane varieties were used: Yuetang 93-159 (YT93-159), Yunzhe 05-51 (YZ05-51), and Yunzhe 08-1609 (YZ08-1609). The experiment was arranged in a randomized complete block design with three biological replicates. Uniform field management practices (fertilization, irrigation, pest and disease control) were consistently applied throughout the sugarcane growing season. The experimental period covered plant cane (PC) and four ratoon cycles (R1 to R4). During the grand growth period (September) of each year, when plants had developed 7–8 stem nodes and 12–13 unfolded leaves, three healthy plants were randomly selected from each plot. Fine absorbing roots were collected from the 0–20 cm soil layer at the plant base and pooled as one biological replicate. Samples were immediately frozen in liquid nitrogen and stored at -80 °C, yielding a total of 45 root samples (3 varieties x 5 years x 3 replicates). Concurrently, cane stem yield (t·ha^-1^) was measured at harvest for subsequent analysis.

### 2.2 RNA extraction, library construction, and high-throughput sequencing

Total RNA was extracted from all root samples using TRIzol^®^ Reagent. The quality of the RNA was rigorously assessed. RNA purity and concentration were determined spectrophotometrically (NanoDrop) and fluorometrically (Qubit), respectively, and RNA integrity was confirmed by agarose gel electrophoresis to ensure clear ribosomal bands without degradation (**Supplementary Figure S1**). Only samples meeting stringent criteria (OD260/280 = 1.8–2.2, OD260/230 ≥ 2.0, 28S:18S ≥ 1.0, and total amount >1 μg) were selected for library construction(**Supplementary Table S1**). Strand-specific mRNA-seq libraries were prepared from 1 μg of total RNA using the Illumina^®^ Stranded mRNA Prep Ligation Kit. The protocol included poly(A) mRNA enrichment using oligo(dT) magnetic beads, followed by fragmentation, double-stranded cDNA synthesis, adapter ligation, and PCR amplification. The resulting libraries were sequenced on the Illumina NovaSeq X Plus platform (PE150) by Majorbio Bio-Pharm Technology Co., Ltd. (Shanghai, China). To ensure data quality, stringent quality control was applied to the raw sequencing data. Using fastp software (version 0.19.5), reads containing adapter sequences, reads with ambiguous bases (N) exceeding 10%, or reads with low-quality bases (Q ≤ 20) exceeding 50% were removed to obtain high-quality clean reads for downstream analyses (Chen, 2023).

### 2.3 Sequence alignment and gene expression quantification

High-quality clean reads were mapped to the sugarcane reference genome (*Saccharum* hybrid ZZ1, version 20231221; https://sugarcane.gxu.edu.cn/scdb) (Chen et al., 2024) using HISAT2 v2.1.0 (Kim et al., 2019). Samples with mapping rates < 50% were considered abnormal and excluded from subsequent analyses to ensure data reliability (Conesa et al., 2016). Gene expression levels were quantified using StringTie v2.1.2 (Pertea et al., 2015), and expression values were reported as fragments per kilobase of transcript per million mapped reads (FPKM) and transcripts per million (TPM). Genes with FPKM > 1 were considered actively expressed (Hart et al., 2013). To assess overall transcriptome similarity and variation among samples, principal component analysis (PCA) and Pearson correlation coefficient (PCC) calculations were performed using R v4.4.3.

### 2.4 Differential expression analysis and functional enrichment

Differentially expressed genes (DEGs) were identified using the DESeq2 package v1.10.1 (Love et al., 2014) based on raw read count matrices. The criteria for DEGs were a false discovery rate (FDR) < 0.05 and |log_2_(Fold Change)|≥ 1. Comparative analyses included pairwise comparisons between consecutive crop cycles for the same variety (e.g., PC vs. R1) and among varieties within the same year. Gene Ontology (GO) enrichment analysis of DEG sets was performed using GOATOOLS (https://pypi.org/project/goatools/), with Benjamini-Hochberg (BH) adjusted *P*-values < 0.05 as the significance threshold.

### 2.5 Mfuzz time-course expression clustering

To capture dynamic gene expression patterns across ratooning cycles, fuzzy C-means clustering was performed using the Mfuzz package v2.6.0 (Singh and Verma, 2022) in R. FPKM expression profiles across five time points (PC–R4) for each variety were Z-score normalized, and genes with low variability (standard deviation, SD < 0.5) were filtered out. For each variety, the optimal fuzzification parameter (m) was first determined using the Dmin function. Subsequently, the optimal cluster number (c) was identified using the minimum centroid distance method based on the established m value. Variety-specific optimal parameters were determined as follows: YT93-159 (c = 25, m = 1.9726), YZ05-51 (c = 20, m = 1.9726), and YZ08-1609 (c = 10, m = 1.9724). Genes with membership scores > 0.5 were assigned to clusters representing distinct temporal expression trends, including sustained upregulation, downregulation, and peak expression patterns.

### 2.6 Weighted gene co-expression network analysis

Co-expression networks were constructed using the WGCNA package v1.63 (Langfelder and Horvath, 2008) based on time-course DEGs for each variety (YT93-159: 702 genes; YZ05-51: 484 genes; YZ08-1609: 681 genes). Prior to network construction, genes were pre-filtered based on mean expression (≥ 1) and coefficient of variation (≤ 0.5). The automatic network construction function blockwiseModules was used. The optimal soft-thresholding powers (β) were set to 6 for YT93-159, 9 for YZ05-51, and 5 for YZ08-1609, which were determined as the lowest powers for which the scale-free topology fit index (R²) reached ≥ 0.85. Other parameters were set as: minimum module size (minModuleSize) = 5, module merge threshold (mergeCutHeight) = 0.25. Unclassified grey modules were excluded. Within significant modules, genes were ranked by module membership (kME) values, with the top 5 selected as hub genes. Cytoscape v3.10.3 (Shannon et al., 2003) was used to visualize interaction networks of hub genes and their associated genes (top 30 by kME ranking with edge weight > 0.02).

### 2.7 Integration of Mfuzz clustering and WGCNA modules

Mfuzz clustering identifies genes with similar expression trends, while WGCNA identifies genes based on expression correlation. To obtain core gene sets identified by both methods, Mfuzz time-course clustering results were integrated with WGCNA co-expression network modules. Statistical significance of gene overlaps between each “Mfuzz cluster-WGCNA module” pair was assessed using hypergeometric tests with BH correction (*P* < 0.05). Overlapping genes with overlap rates > 45% were selected to construct integrated modules for subsequent functional analysis.

### 2.8 qRT-PCR validation

To validate RNA-seq data reliability, seven candidate hub genes were selected for quantitative real-time PCR (qRT-PCR) verification. Total RNA was reverse transcribed to cDNA using HiScript^®^ III All-in-one RT SuperMix Perfect for qPCR kit (Vazyme, R333-01). Amplification was performed on a StepOnePlus Real-Time PCR System (Applied Biosystems) with three technical replicates per sample. Gene-specific primers (**Supplementary Table S2**) were designed using NCBI Primer-BLAST. PCR conditions were: 94°C for 4 min; 35 cycles of 94°C for 30 s, 60°C for 30 s, 72°C for 30 s; final extension at 72°C for 7 min. Relative expression was calculated using the comparative Ct (2^-ΔΔCt^) method (Livak and Schmittgen, 2001).

## 3 Results

### 3.1 Sugarcane yield significantly decreased at R3 stage

**Figure 1:**
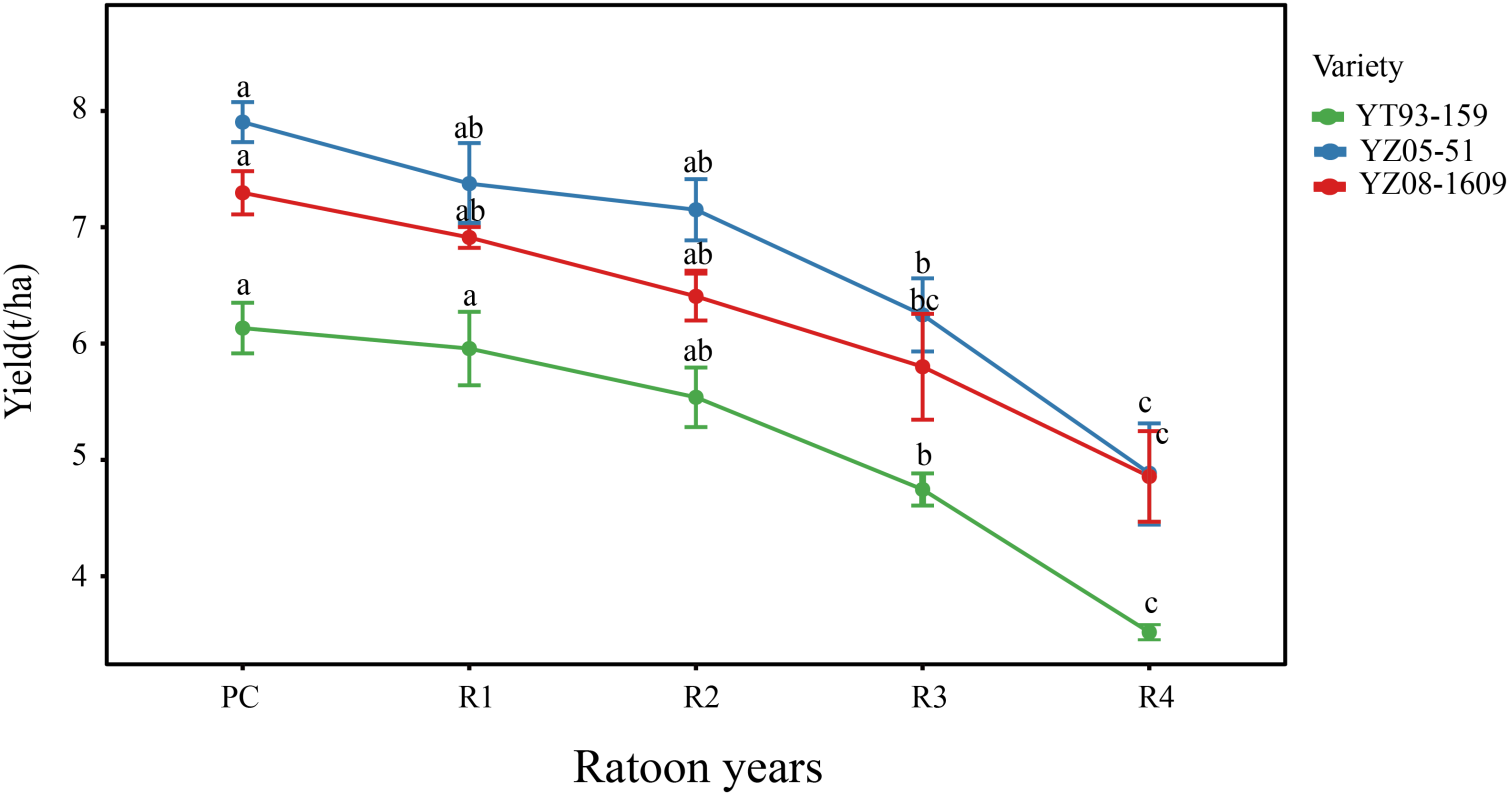
Yield differences among major sugarcane varieties across different ratoon planting years. PC, plant cane; R1–R4, first to fourth ratoon years. Data are presented as mean ± SE (n = 6). Different lowercase letters indicate significant differences among years within the same variety (*P* < 0.05).

To identify the critical period of ratooning decline, cane yield were continuously monitored across three varieties from PC to R4 (**Figure 1**). Results showed that yields of all varieties exhibited a declining trend with increasing ratooning years. No significant differences in yield were observed among varieties during the PC, R1, and R2 stages (*P* > 0.05). From R2 to R3, all three varieties exhibited their first significant yield decline (*P* < 0.05), with reductions of 14.3%, 12.64%, and 9.45% for YT93-159, YZ05-51, and YZ08-1609, respectively. This declining trend continued into the R4 stage. These data indicated that the R2 to R3 transition represented the initial phase of yield decline.

### 3.2 Transcriptome sequencing and global expression dynamics analysis

To investigate the molecular mechanisms underlying the critical turning point of ratooning decline, transcriptome sequencing was performed on 45 root samples from three sugarcane varieties across five periods. After stringent quality control, a total of 449.59 Gb of high-quality clean data were obtained, with approximately 67.33 million clean reads per sample and Q30 base percentages ≥ 97.27% (**Supplementary Table S3**). Through alignment analysis, four samples were excluded due to mapping rates below the 50% threshold (one replicate each for YZ05–51 in R1 and R2, and YT93–159 in R2 and R4), resulting in a final dataset of 41 high-quality samples for subsequent differential expression and functional enrichment analyses.

Principal component analysis (PCA) was performed to assess global transcriptome dynamics of 41 qualified samples throughout the ratooning decline process. PCA results showed clear separation by genotype with tight clustering of biological replicates within varieties, indicating high reliability of experimental design and sequencing quality (**Supplementary Figure S2**). Importantly, when analyzed by variety, all samples displayed continuous gradient distribution along PC1 (variance contribution 22.13%–38.64%) from PC to R4 (**Supplementary Figures S3A–C**). Sample dispersion increased markedly from the R2 stage onwards. Additionally, Pearson correlation coefficient (PCC) analysis identified R2 as a transcriptomic bifurcation point where inter-variety correlations exhibited contrasting patterns (**Supplementary Figures S3D–F**): correlations involving YZ05–51 reached their minimum values at R2 (PCC: YZ08–1609 vs. YZ05-51 = 0.43; YZ05–51 vs. YT93-159 = 0.44), while YZ08–1609 and YT93–159 displayed their maximum correlation at this stage (PCC = 0.79).

### 3.3 Gene expression dynamics reveal R2–R3 as the key turning point for transcriptional reprogramming

**Figure 2:**
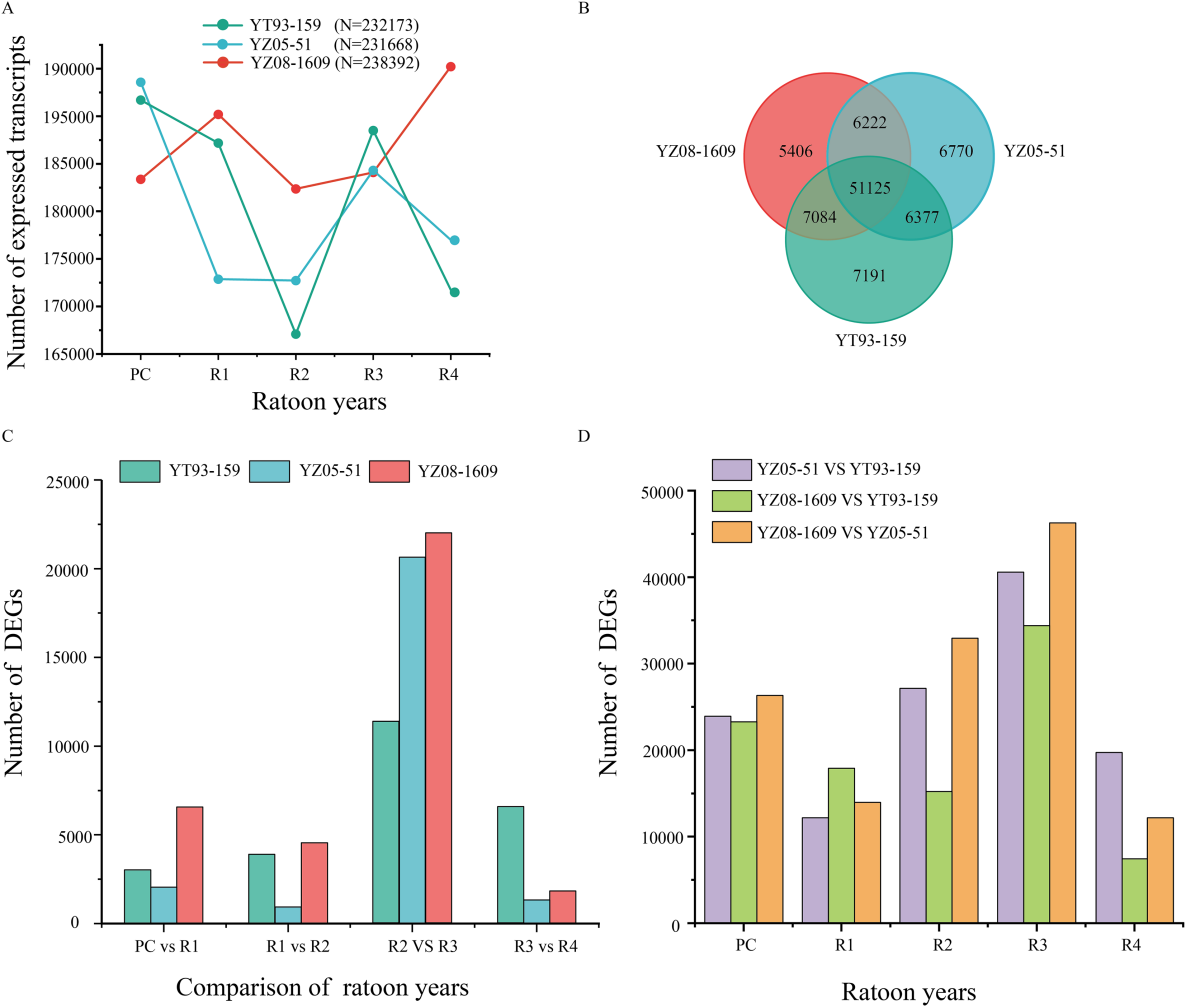
Transcriptome expression pattern analysis of three sugarcane varieties during different ratoon years. **(A)** Line charts showing the dynamic changes in the total number of expressed transcripts from PC to R4 in YT93-159, YZ05-51, and YZ08-1609. **(B)** Venn diagram of the shared and unique expressed genes among the three varieties. Each color represents one cultivar. **(C)** Number of differentially expressed genes (DEGs) between adjacent ratoon years within each variety. **(D)** Number of DEGs identified between varieties at each ratoon year.

Temporal analysis of root transcriptomes from three sugarcane varieties indicated that ratooning decline is a biological process precisely regulated by complex regulatory networks. Throughout the ratooning cycle, 232,173, 231,668, and 238,392 expressed transcripts were detected in the roots of the three varieties, respectively (**Figure 2A**). Among these, 51,125 genes were stably co-expressed across all samples (FPKM > 1), constituting the “core transcriptome” that maintains basic root physiological functions (**Figure 2B**).The study revealed that the total number of transcripts in each variety reached its lowest point at R2, followed by a significant rebound at R3. This expression dynamic pattern suggests that R2 represents a physiological homeostatic plateau, whereas R3 marks a critical transition point initiating large-scale gene expression reprogramming. Differential gene expression analysis provided direct evidence for this critical transition. From R2 to R3, there was an explosive increase in the number of DEGs, reaching 11,348, 20,638, and 21,977 in the three varieties, respectively (**Figure 2C**), far exceeding the changes observed in other adjacent periods. This dramatic transcriptome remodeling was also reflected in inter-varietal differences. At R3, transcriptome divergence between varieties peaked, with the number of DEGs between YZ08–1609 and YZ05–51 reaching as high as 46,280 (**Figure 2D**). These results indicate that varieties with different genetic backgrounds adopt highly differentiated molecular response strategies upon entering the critical stage of ratooning decline.

### 3.4 Time-course expression clustering reveals conserved regulatory modules

**Figure 3:**
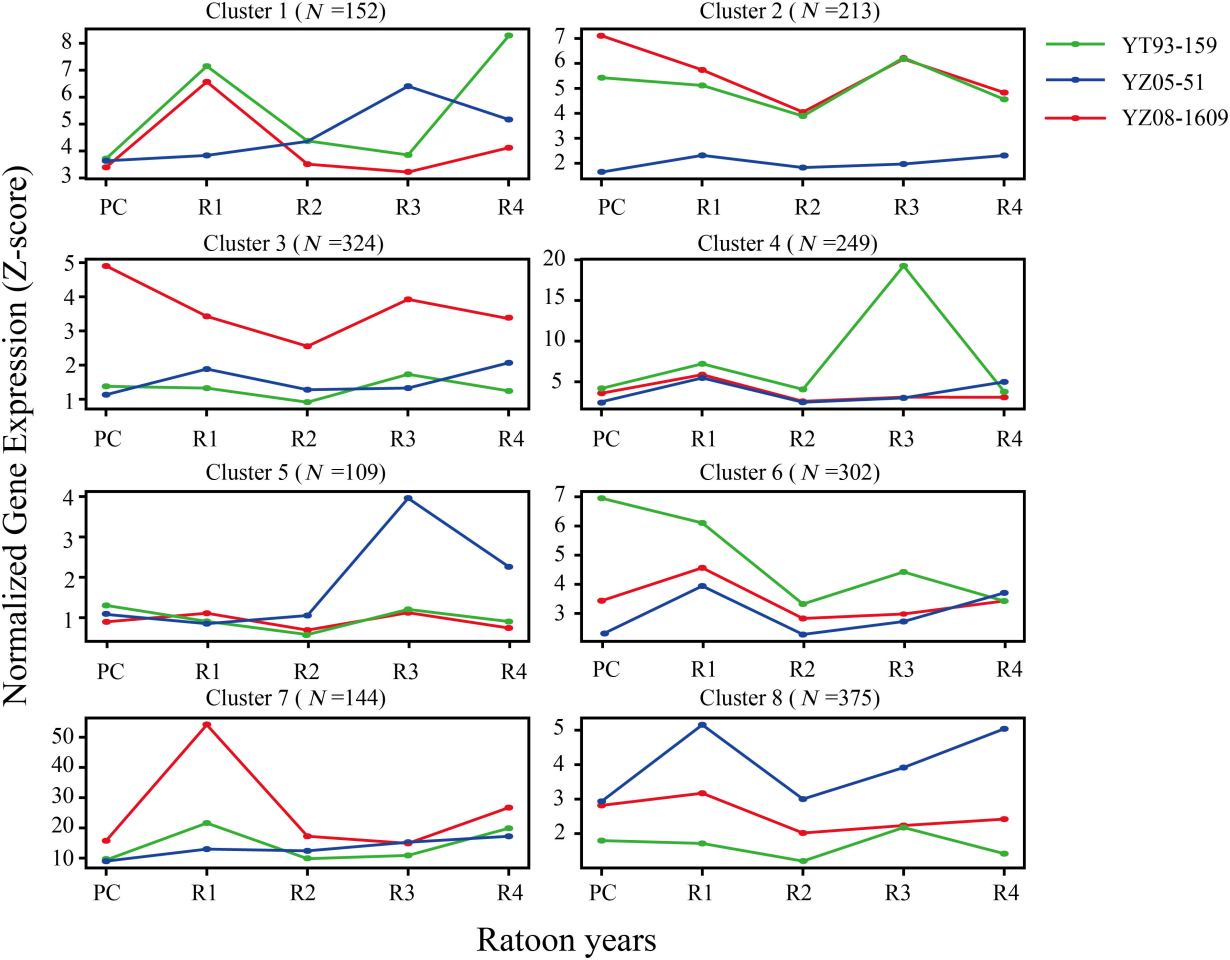
Gene expression pattern clustering analysis based on ratoon years. Eight distinct expression clusters are shown, each representing a unique trend of gene expression. The number in parentheses (N) indicates the number of genes in each cluster. Curves in different colors represent the varieties YT93-159, YZ05-51, and YZ08-1609, connecting the average Z-score normalized expression levels of genes across ratoon years (PC, R1–R4).

To investigate dynamic gene expression patterns during sugarcane ratooning decline, Mfuzz time-course clustering was performed on transcriptome data from three varieties. Genes exhibiting similar expression trends across all varieties were considered to constitute conserved regulatory modules that drive ratooning decline. Based on this criterion, screening and intersection analysis of expression profiles identified a conserved set of 1,869 genes exhibiting similar temporal expression patterns across all varieties. These genes were further partitioned into eight subclusters with distinct expression patterns (Subclusters 1–8; **Figure 3**) using hierarchical clustering.

**Figure 4:**
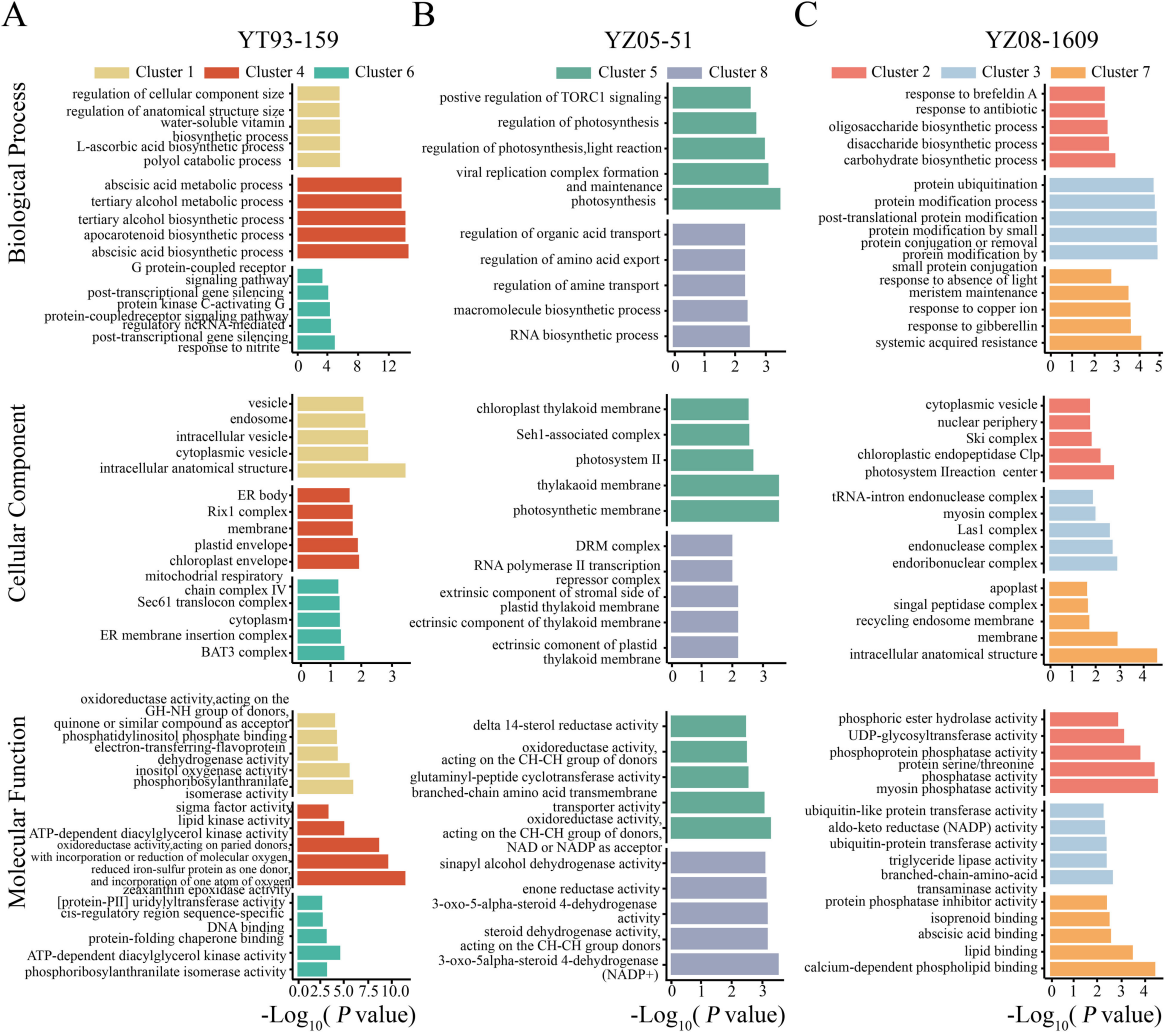
GO enrichment analysis of gene expression clusters in three sugarcane varieties. GO enrichment results for YT93-159 **(A)**, YZ05-51 **(B)**, and YZ08-1609 **(C)**, including the biological process, cellular component, and molecular function categories. Different colors represent gene sets from different clusters. The X-axis represents -log_10_(*P*-value), and the Y-axis shows the enriched GO terms.

To elucidate the underlying biological functions, GO enrichment analysis was performed for each subcluster (**Figure 4**). The three varieties exhibited distinct molecular regulatory strategies during the R2-R3 transition period, which correlated closely with their yield performance at R3. YT93–159 showed the largest yield decline (14.3%), with molecular responses dominated by passive emergency stress signaling. Genes in Cluster 4 were significantly upregulated at R3 and highly enriched in abscisic acid (ABA) biosynthesis, carotenoid metabolism, and zeaxanthin epoxidase activity—hallmarks of plant emergency responses to severe oxidative stress and photoinhibition—indicating pronounced oxidative damage in YT93–159 at R3. Cluster 1 was enriched in polyol catabolic processes and oxidoreductase activity, potentially involved in osmotic regulation and maintenance of redox homeostasis. Cluster 6 showed enrichment in G-protein coupled receptor (GPCR) signaling and non-coding RNA-mediated post-transcriptional gene silencing, suggesting activation of stress signal perception mechanisms.

YZ05–51 exhibited a moderate yield decline (11.8%), with molecular responses emphasizing maintenance of basal metabolic activity. Genes in Cluster 5 were significantly upregulated at R3, enriched in cellular respiration, transmembrane transport, and carbohydrate catabolism, indicating prioritized energy supply and material transport in this variety. Cluster 8 was enriched in RNA biosynthesis and transcriptional regulation. YZ08–1609 showed the smallest yield reduction (9.45%), characterized by active defense and cellular homeostasis maintenance. Cluster 7 was enriched in systemic acquired resistance (SAR), copper ion response, and plant hormone signaling, reflecting activation of whole-plant immune defenses effectively suppressing pathogen accumulation and oxidative damage. Cluster 3 displayed enrichment in post-translational protein modification and nucleic acid processing, indicative of higher cellular homeostasis. Cluster 2 was enriched in carbohydrate biosynthesis and protein phosphatase activity, suggesting this variety sustained normal carbon metabolism and signaling functions.

### 3.5 Integration of WGCNA and Mfuzz identifies core functional modules

**Figure 5:**
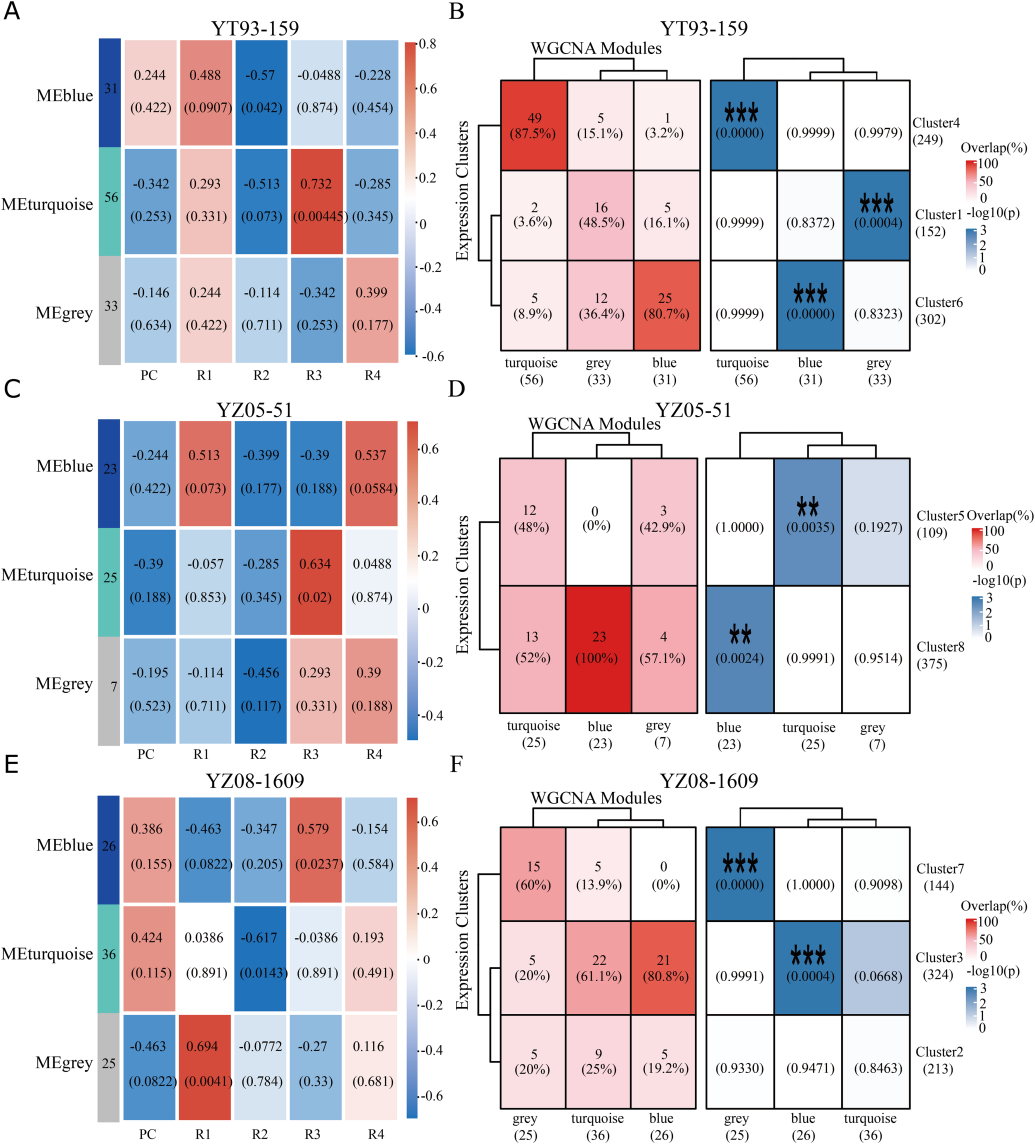
WGCNA and overlap analysis with expression clusters. **(A, C, E)** Heatmaps showing the correlation between gene modules and ratoon years in the three sugarcane varieties YT93-159, YZ05-51, and YZ08-1609. Each row represents a module eigengene (ME), and each column represents a ratoon year. Numbers in the squares indicate the correlation coefficient and the corresponding *P*-value (in parentheses). The color bar on the right indicates the strength and direction of correlation (red, positive; blue, negative). **(B, D, F)** Overlap and correlation analysis between WGCNA modules and gene expression clusters. Numbers in the squares indicate the number and percentage of genes shared between the module and cluster. Colors and asterisks denote the significance level of the correlation (***P < 0.001,**P < 0.01).

To further explore gene interactions, weighted gene co-expression network analysis (WGCNA) was performed on variety-dominant gene sets from the time-course clustering. Co-expression networks containing 702, 484, and 681 genes were constructed for YT93-159, YZ05-51, and YZ08-1609, respectively (**Figures 5A, C, E**). After excluding gray (invalid) modules, two main co-expression modules were identified per variety. The number of genes assigned to these key modules was as follows: in YT93-159, the turquoise and blue modules contained 56 and 31 genes, respectively; in YZ05-51, they contained 25 and 23 genes; and in YT08-1609, they contained 36 and 26 genes. Module-ratooning stage correlation analysis revealed the following patterns. In YT93-159, the turquoise module showed significant positive correlation with R3 (Pearson’s r = 0.732), while the blue module was negatively correlated with R2 (r = -0.570). In YZ05-51, the turquoise module exhibited positive correlation with R3 (r = 0.634). In YZ08-1609, the turquoise module was negatively correlated with R2 (r = -0.617), whereas the blue module showed positive correlation with R2 (r = 0.579).

Gene overlap analysis between Mfuzz clusters and WGCNA modules revealed high concordance across all three varieties (**Figures 5B, D, F**). For instance, in YT93-159, 87.5% of turquoise module genes overlapped with Cluster 4; in YZ05-51, 100% of blue module genes overlapped with Cluster 8; in YZ08-1609, 80.8% of blue module genes overlapped with Cluster 3. Genes identified in both WGCNA modules and Mfuzz clusters were designated as core functional genes associated with ratooning decline. Module-cluster pairs showing both high gene overlap (> 45%) and significant correlation with ratooning stages (|r| > 0.5, *P* < 0.01) were integrated to define core functional modules. Five integrated modules were identified: two in YT93-159 (turquoise module overlapping with Cluster 4 and blue module with Cluster 6), two in YZ05-51 (turquoise module with Cluster 5 and blue module with Cluster 8), and one in YZ08-1609 (blue module with Cluster 3).

GO enrichment analysis of integrated WGCNA modules further clarified the functional differentiation among varieties during ratoon decline (**Supplementary Figure S4**). The enzymatic activities enriched within each module aligned well with the overall strategies suggested by their temporal clustering. In YT93-159, two modules were respectively associated with stress signaling and energy supply. The YT93-Cluster4-Turquoise module was enriched in zeaxanthin epoxidase activity—a key enzyme in ABA biosynthesis—and oxidoreductase activity, indicating initiation of stress responses through the ABA pathway and regulation of redox status. The YT93-Cluster6-Blue module was enriched in mitochondrial respiratory chain complex IV and nucleoside diphosphate kinase activities, reflecting enhanced energy metabolism and nucleotide synthesis to support stress responses.

In YZ05-51, module functions centered on maintenance of basal metabolism. The YZ05-Cluster5-Turquoise module showed enrichment of acetolactate synthase activity (a key enzyme in branched-chain amino acid synthesis) and oxidoreductase activity, underscoring its role in sustaining amino acid biosynthesis and redox balance. The YZ05-Cluster8-Blue module was enriched in 3-oxo-5α-steroid 4-dehydrogenase and enoyl reductase activities, reflecting potential functions in hormone metabolism and reduction reactions, jointly supporting metabolic stability under stress. For YZ08-1609, modules were linked to stress mitigation and protein homeostasis. The YZ08-Cluster3-Blue module was enriched in NADP-dependent aldehyde reductase and ubiquitin-protein transferase activities, suggesting coordinated detoxification mechanisms and protein degradation pathways to maintain redox homeostasis and protein quality control, thereby enhancing cellular adaptation to sustained stress.

### 3.6 Identification and validation of candidate genes

Based on module membership (kME), the top five genes with the highest kME values were selected from each of the five integrated modules as hub genes, yielding a total of 25 hub genes (**Supplementary Table S4**). To visualize gene interaction networks within each module, high-weight gene pairs were displayed using Cytoscape (**Supplementary Figure S5**). Notably, 15 of these hub genes (60%) showed peak expression at the R3 stage across varieties, underscoring the importance of this stage as a critical transition point in ratooning decline (**Supplementary Table S5**). The remaining 10 hub genes exhibited peak expression at earlier stages (R1), suggesting stage-specific regulatory functions throughout the ratooning progression.

For example, in the YT93-Cluster4-Turquoise module (n = 49), which showed significant correlation with R3, five hub genes were identified. Among these, three were *GOLS2* homologs (*MSTRG.86658, ROC-So-Chr01D0009140, MSTRG.160763*) encoding galactinol synthase 2, a rate-limiting enzyme in raffinose family oligosaccharide (RFO) biosynthesis involved in osmotic regulation (Yan et al., 2022). The other two hub genes encoded the plastid-localized protein *PAM68* (*ROC-Rec-Chr01A0053830*), which functions in photosystem maintenance, and *MHZ4* (*ROC-So-Chr02D0008080*), which is involved in ABA and auxin signaling integration (Xiong et al., 2025; Zhang et al., 2024a). All five genes showed significant upregulation at the R3 stage, consistent with their roles in stress responses during ratooning decline.

The YZ05-Cluster5-Turquoise module (n = 12) from YZ05-51, strongly correlated with R3, contained five hub genes involved in stress responses. These included genes encoding thiosulfate sulfurtransferases *STR16* and *STR18* (*MSTRG.161669*, *YZ-So-Chr07D0004470*) for oxidative stress and sulfur homeostasis (Luo et al., 2025), a Bowman-Birk-type trypsin inhibitor (*BBI*; *MSTRG.91796*) for disease resistance (Wilson and Chen, 1983), GEM-like protein 4 (*GLP4*) (*YZ-Rec-Chr01A0010450*) for developmental regulation and multi-stress responses (Bernier and Berna, 2001), and cysteine-rich transmembrane module protein 3 (*CYSTM3*; *ROC-So-Chr05A0009160*) for abiotic stress tolerance (Xu et al., 2018).

The YZ08-Cluster3-Blue module (n = 21) from YZ08-1609, strongly correlated with R3, contained five hub genes involved in protein homeostasis and cellular regulation. These included genes encoding *UPS* components E3 ubiquitin-protein ligase *ATL41* (*YZ-So-Chr02A0009970*) and an F-box protein (*YZ-So-Chr07A0006350*) for protein quality control (Horn-Ghetko et al., 2021), the immune regulator *ITN1* (*MSTRG.172238*) for *TNL*-mediated defense signaling (Wu et al., 2025), the mitochondrial assembly factor *BCS1* (*YZ-Ss-Chr06B0019600*) for respiratory chain function (Pan et al., 2023), and the transcription factor *ZFP1* (*MSTRG.21125)* for stress-responsive gene regulation (Gosztyla et al., 2024).

**Figure 6:**
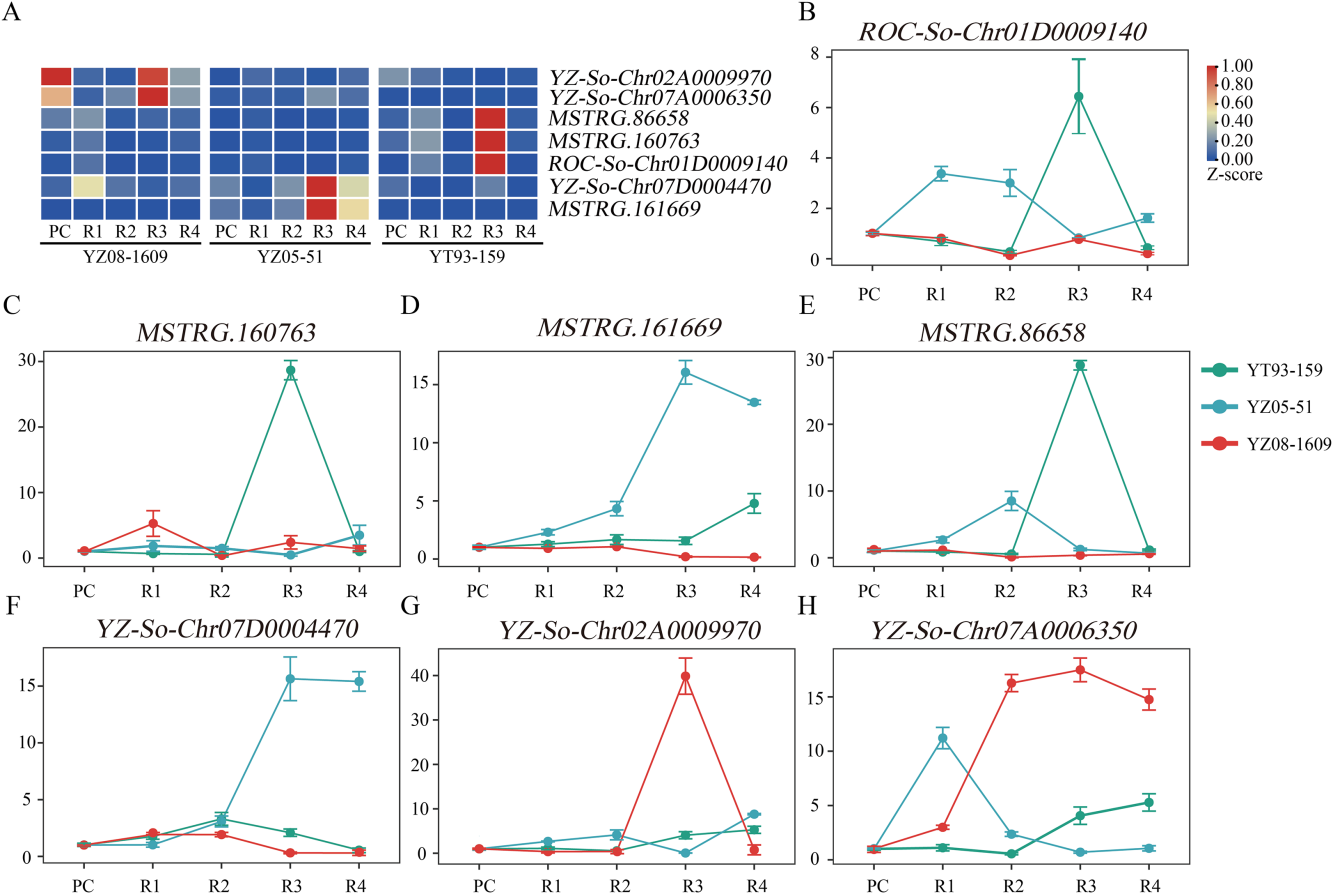
qRT-PCR validation and RNA-seq expression pattern analysis of candidate genes. **(A)** Heatmap showing RNA-seq expression patterns of seven candidate genes in three sugarcane varieties (YT93-159, YZ05-51, and YZ08-1609) across ratoon years (PC, R1–R4). Expression values are Z-score normalized (blue, low expression; red, high expression). **(B–H)** qRT-PCR validation of candidate gene expression across ratoon years. Lines represent YT93-159 (green), YZ05-51 (blue), and YZ08-1609 (red). Data are mean ± SE (n = 3 biological replicates).

To validate the reliability of RNA-seq data, seven representative hub genes were selected for quantitative real-time PCR (qRT-PCR) analysis using the ubiquitin gene (*UBQ*) as an internal reference (primer sequences are provided in **Supplementary Table S2**). The relative expression patterns obtained from qRT-PCR were highly consistent with the RNA-seq results (**Figure 6**), confirming the credibility and accuracy of the transcriptomic data.

## 4 Discussion

### 4.1 R2–R3 as the critical turning point for sugarcane ratooning decline

By integrating five-year field yield data with root temporal transcriptome profiles, this study identified the R2–R3 transition as a critical turning point in sugarcane ratooning decline. This conclusion is supported by convergent evidence from both phenotypic and transcriptomic analyses: all three varieties exhibited their first significant yield reduction at R3 (9.45%–14.3%), preceded by systematic transcriptional reprogramming(**Figure 1**). At R2, transcriptomic instability began to emerge, characterized by the lowest transcript abundance (**Figure 2A**), increased dispersion in principal component analysis (**Supplementary Figures S3A–C**), and differentiation in inter-varietal correlation patterns (**Supplementary Figures S3D–F**). This destabilization phase peaked at R3, marked by an explosive increase in differentially expressed genes (DEGs) (**Figure 2C**) and maximized transcriptomic divergence among varieties (**Figure 2D**).

The pronounced “transcriptomic bottleneck” at R2, occurring just before the initial yield decline at R3, may signify a key physiological reset. We hypothesize that by R2, cumulative stress exceeded the root system’s capacity to maintain homeostasis, triggering a global, transient transcriptional shutdown to conserve energy and mitigate oxidative damage. This bottleneck represents a shift from a growth-sustaining homeostatic state to a survival mode prioritizing energy preservation. The subsequent transcriptomic rebound at R3 reflects the initiation of costly adaptive reprogramming. The observed yield loss is likely a direct physiological consequence of this resource reallocation, whereby considerable metabolic resources invested in transcriptional remodeling divert energy otherwise dedicated to sucrose accumulation and stalk development. This mechanistically explains the observed reductions in single stalk weight and ratoon tiller number.

### 4.2 Multi-process synergistic dysregulation as the molecular basis of ratooning decline

In-depth transcriptomic analysis of root tissues at the R3 stage revealed that ratoon decline manifests at the molecular level as a systemic dysregulation involving multiple key biological processes, including energy metabolism, signal transduction, protein homeostasis, and defense responses. Despite genetic background differences, all three varieties exhibited significant reprogramming of these core processes, forming an interconnected network of functional disruption (**Figure 4**; **Supplementary Figure S4**). This coordinated multisystem activation supports the view that ratoon decline reflects a resource reallocation trade-off strategy in roots under multiple combined stresses (Hou and Xu, 2025; Simas et al., 2025).

On the one hand, enrichment of energy metabolism pathways such as oxidative phosphorylation and carbohydrate catabolism reflects root energy mobilization to meet increased demands for defense, repair, and maintenance (Heinemann and Hildebrandt, 2021; Zhang et al., 2024b; Zou et al., 2024). This is consistent with the energy crisis characteristic widely observed in perennial plants undergoing senescence or stress (González-Paleo et al., 2016; Schwarzländer and Finkemeier, 2013; Tunc and von Wirén, 2025). However, under limited energy supply conditions, such compensatory activation is unsustainable and eventually leads to energy depletion. Energy insufficiency could impair active nutrient uptake and growth capacity of roots, thereby affecting axillary bud germination and the supply of carbohydrates and nutrients to tillers (Li et al., 2024a, 2024; Zhang et al., 2024b; Zhao, 2024). This mechanism temporally correlates with the significant yield reductions (9.45%–14.3%, **Figure 1**) first observed at the R3 stage in the three varieties. On the other hand, widespread activation of protein quality control systems, including the ubiquitin-proteasome pathway and endoplasmic reticulum protein processing, indicates severe challenges to protein homeostasis in root cells at R3 (Liu and Howell, 2010; Sun et al., 2021). This may result from accumulation of misfolded proteins under stress, triggering energy-intensive clearance mechanisms (Ogawa-Ohnishi et al., 2022; Shin et al., 2017). Overactivation of protein degradation not only consumes large amounts of ATP but also disrupts normal cellular metabolism, reducing overall root functional efficiency (Hellmann and Estelle, 2002; Huang et al., 2010; Rojas-Pierce and Bednarek, 2025). Simultaneously, persistent activation of defense response pathways such as phenylpropanoid biosynthesis and plant-pathogen interactions, while beneficial for short-term stress adaptation, inevitably consumes carbon skeletons and energy required for growth over the long term. This observation aligns with the classical “growth-defense trade-off” theory (Guayazán-Palacios and Steinbrenner, 2025; Li et al., 2021; Ramirez-Prado et al., 2018; Zrimec et al., 2025). Increased synthesis of defense-related secondary metabolites may reduce sucrose transport and accumulation in stems, potentially contributing to the decrease in single stalk weight.

Therefore, ratoon decline is unlikely driven by a single factor; instead, it results from a disruption in the coordination among core systems, such as energy supply, protein homeostasis, and defense responses, under conditions of prolonged metabolic load and stress. Molecular-level dysregulation may impair key physiological functions of roots including nutrient uptake, growth vigor, and metabolic efficiency, ultimately leading to decreased axillary bud germination, reduced tiller number, and lowered single stalk weight. These phenotypic changes coincide with the notable yield decline observed in the field (**Figure 1**). This cascade from molecular perturbation to phenotypic deterioration reveals the systemic nature of ratoon decline and provides important insights into its complex mechanisms.

### 4.3 Variety-specific hub genes reveal differential response strategies to ratooning decline

One of the most significant findings of this study is that different varieties exhibited distinct regulatory strategies when facing common ratooning decline pressure. Through identification and functional analysis of hub genes, we revealed that YT93-159, YZ05-51, and YZ08–1609 relied on three different dominant pathways: osmotic regulation, oxidative stress defense, and protein quality control, respectively. The three *GOLS* genes in YT93–159 were co-upregulated at R3, with increases up to 102.21%. As a rate-limiting enzyme in raffinose family oligosaccharides (RFOs) biosynthesis, *GOLS* mitigates systemic disorder through dual mechanisms: RFOs serve as carbon reserves for energy reallocation while maintaining cellular homeostasis via osmotic adjustment and reactive oxygen species (ROS) scavenging (Li et al., 2017; Taji et al., 2002; Yan et al., 2022). This response is conserved in grasses. For example, wheat *TaGolS* confers drought and ER stress tolerance (Singh et al., 2024). However, RFO synthesis is carbon and energy intensive (Martins et al., 2022; Noronha et al., 2021; Salvi et al., 2021). Under energy-limited conditions at R3, diversion of carbon to RFOs may exacerbate energy deficiency, reflecting a growth-survival trade-off (dos Santos and Vieira, 2020; Karner et al., 2004).

In YZ05-51, two *STR* genes were co-upregulated at R3. *STR*s aid stress tolerance through dual mechanisms. They support Fe-S cluster biogenesis to maintain mitochondrial energy metabolism, which is consistent with enriched oxidative phosphorylation pathways at R3 (Braymer and Lill, 2017; Lill and Freibert, 2020; Rydz et al., 2021; Schilke et al., 2006). On the other hand, *STR* catalyzes protein persulfidation modification (forming -SSH groups on critical cysteine residues), which reversibly protects key enzymes from oxidative damage caused by reactive oxygen species (Papenbrock and Schmidt, 2000).Rice *OsSTR1* is similarly induced under cadmium stress and localizes to mitochondria (Guretzki and Papenbrock, 2011), supporting the conserved role of *STR* in maintaining redox homeostasis under stress. While this dual protection reduces energy expenditure by preserving existing proteins, *de novo* Fe-S cluster synthesis consumes ATP, potentially competing with other metabolic processes under energy scarcity (Marszalek et al., 2024; Tong et al., 2018; Want and D'Autréaux, 2024). YZ08–1609 showed significant upregulation of *UPS*-related genes at R3, including E3 ubiquitin ligases and F-box proteins. These genes form functional networks where E3 ubiquitin ligases determine substrate specificity, F-box proteins mediate specific ubiquitination, and immune regulatory factors such as *ITN1* balance defense responses with protein degradation (Knop et al., 2021; Lan and Miao, 2019; Skaar et al., 2013; Wu et al., 2023). The *UPS* prevents toxic protein aggregation through selective degradation of damaged proteins and temporally modulates defense gene expression by differentially degrading stress-related transcription factors such as *NPR1* and *JAZ* inhibitors (Nagels Durand et al., 2016; Wu et al., 2024). However, the high energy demands of *UPS* may drive compensatory activation of oxidative phosphorylation at R3, suggesting that YZ08–1609 prioritizes energy allocation to proteostasis through metabolic reprogramming.

Sugarcane varieties employ distinct molecular strategies—osmotolerance (*GOLS*), oxidative defense (*STR*), and protein homeostasis (*UPS*)—to mitigate ratooning decline, yet all face metabolic trade-offs under root energy constraints. The specific upregulation of these genes delineates variety-specific responses. Their documented roles in related stress responses support the biological relevance of our findings and position them as key candidates for functional characterization. This multi-genotype comparison systematically uncovers these specialized strategies, moving beyond single-variety studies to provide a genotype-aware blueprint for targeted breeding against ratooning decline.

## 5 Limitations and future directions

However, it is important to note that the conclusions of this study are primarily based on transcriptome data, reflecting regulatory patterns at the gene expression level, but have not yet been directly validated through functional experiments to establish causality. Further confirmation of the direct roles of these genes and mechanisms will require genetic functional validation, transgenic approaches, and phenotypic analyses. Additionally, transcriptome profiling cannot capture dynamic changes at the protein level or metabolic activities. Future studies should integrate multi-omics approaches to comprehensively elucidate the mechanisms underlying ratoon decline.

## 6 Conclusions

This study elucidated the molecular mechanisms underlying sugarcane ratooning decline by integrating five-year field trials with root transcriptome analysis. The third ratoon year (R3) was identified as a critical transition period, characterized by significant yield reduction and extensive global transcriptional reprogramming in roots. Comparative transcriptomics revealed coordinated dysregulation of energy metabolism, signal transduction, protein homeostasis, and defense pathways across all varieties at R3, representing conserved molecular features of decline. However, distinct variety-specific regulatory strategies were evident: YT93–159 activated *GOLS*-mediated osmotic protection, YZ05–51 enhanced *STR*-dependent redox homeostasis, and YZ08–1609 upregulated *UPS*-related genes for protein quality control. These findings highlight the determinant role of genetic background in decline progression and underscore the necessity of variety-targeted approaches for precision breeding. Co-expression network analysis identified 25 core genes, with 15 conserved hub genes peaking at R3, providing priority candidates for marker development and functional characterization. The study established an integrative analytical framework encompassing critical periods, conserved pathways, variety-specific mechanisms, and candidate genes, offering a theoretical foundation and genetic resources for targeted mitigation of ratooning decline in sugarcane.

## Data Availability

The datasets presented in this study can be found in online repositories. The raw reads have been deposited in the Genome Sequence Archive (GSA, https://ngdc.cncb.ac.cn/gsa/) under accession number CRA032661. All other generated datasets are provided within the article and **Supplementary Information** files.
