# Supplementary material for: Transcriptome time-course analysis unravels the regulatory networks governing ratooning decline in sugarcane
Source: Front Plant Sci. 2026 Jan 12;16:1739058. doi: 10.3389/fpls.2025.1739058 (PMC12832970; doi:10.3389/fpls.2025.1739058)
Supplement: Supplementary file 1 [file DataSheet1.docx]

Transcriptome Time-Course Analysis unravels the regulatory networks governing ratooning decline in sugarcane

**Supporting Figure S1- S5**

Figure S1. Assessment of RNA integrity by agarose gel electrophoresis.

Figure S2. Principal component analysis of all samples.

Figure S3. Principal component analysis of three sugarcane varieties respectively, and Pearson correlation coefficient analysis between each pair of varieties.

Figure S4. GO enrichment analysis results of five key gene co-expression modules.

Figure S5. Gene co-expression correlation networks of five key gene co-expression modules.


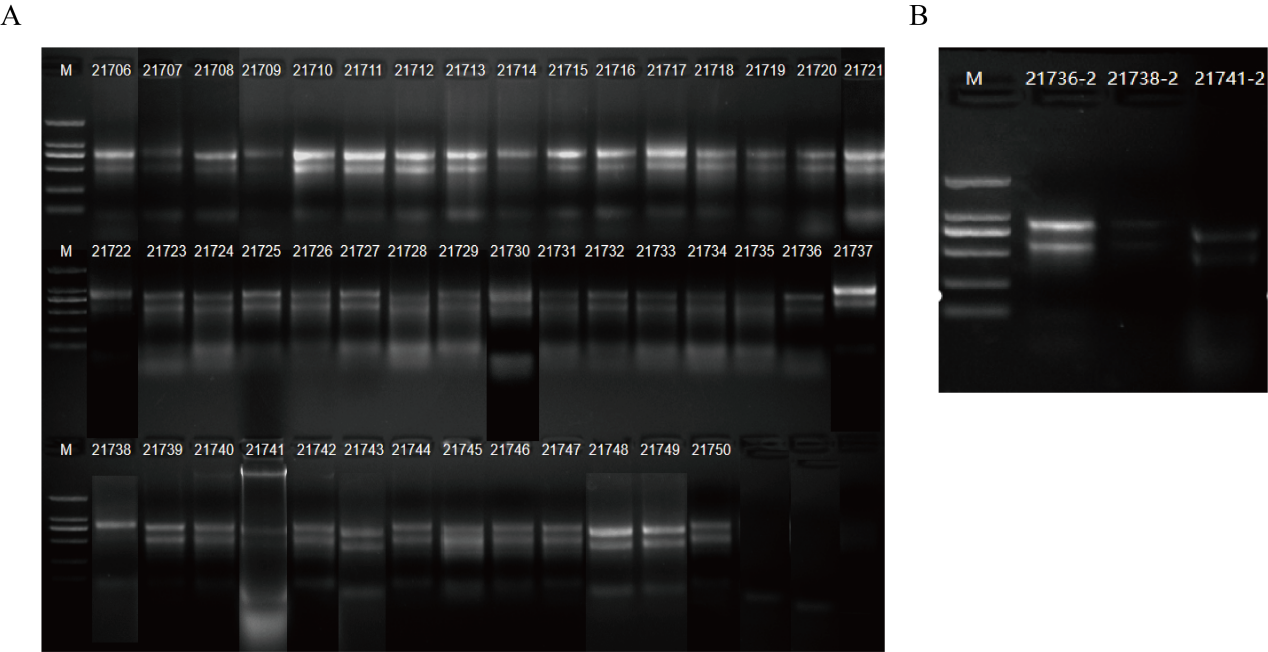


Figure S1. Assessment of RNA integrity by agarose gel electrophoresis. A: Agarose gel electrophoresis of total RNA from all initial samples (IDs 21706–21750). The presence of sharp, distinct ribosomal RNA bands (28S and 18S) confirms high RNA integrity without degradation. B: Repeat electrophoresis of total RNA from samples that showed suboptimal quality or suspected degradation in the initial run (A). Re-tested samples (identified with the suffix "-2") include 21736-2, 21738-2, and 21741-2.M: DNA molecular weight marker (RNA Ladders are more common for this application, but DNA markers can be used for approximate size estimation).


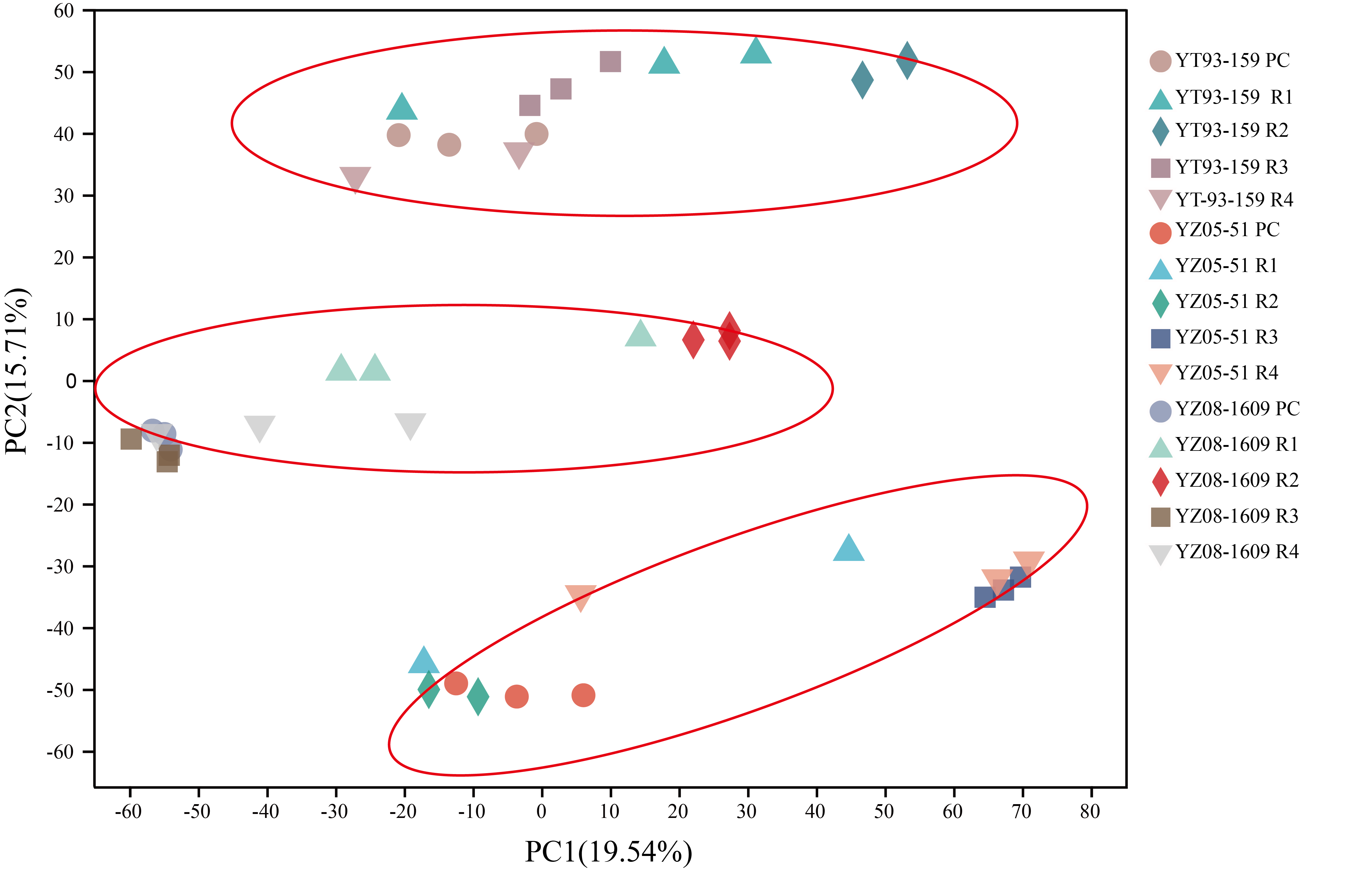


Figure S2. Principal component analysis of all samples. Different shapes represent different ratooning stages (plant cane and ratoon years 1–4), and different colors distinguish samples from the three sugarcane varieties at each stage. Red ellipses indicate clustering patterns.


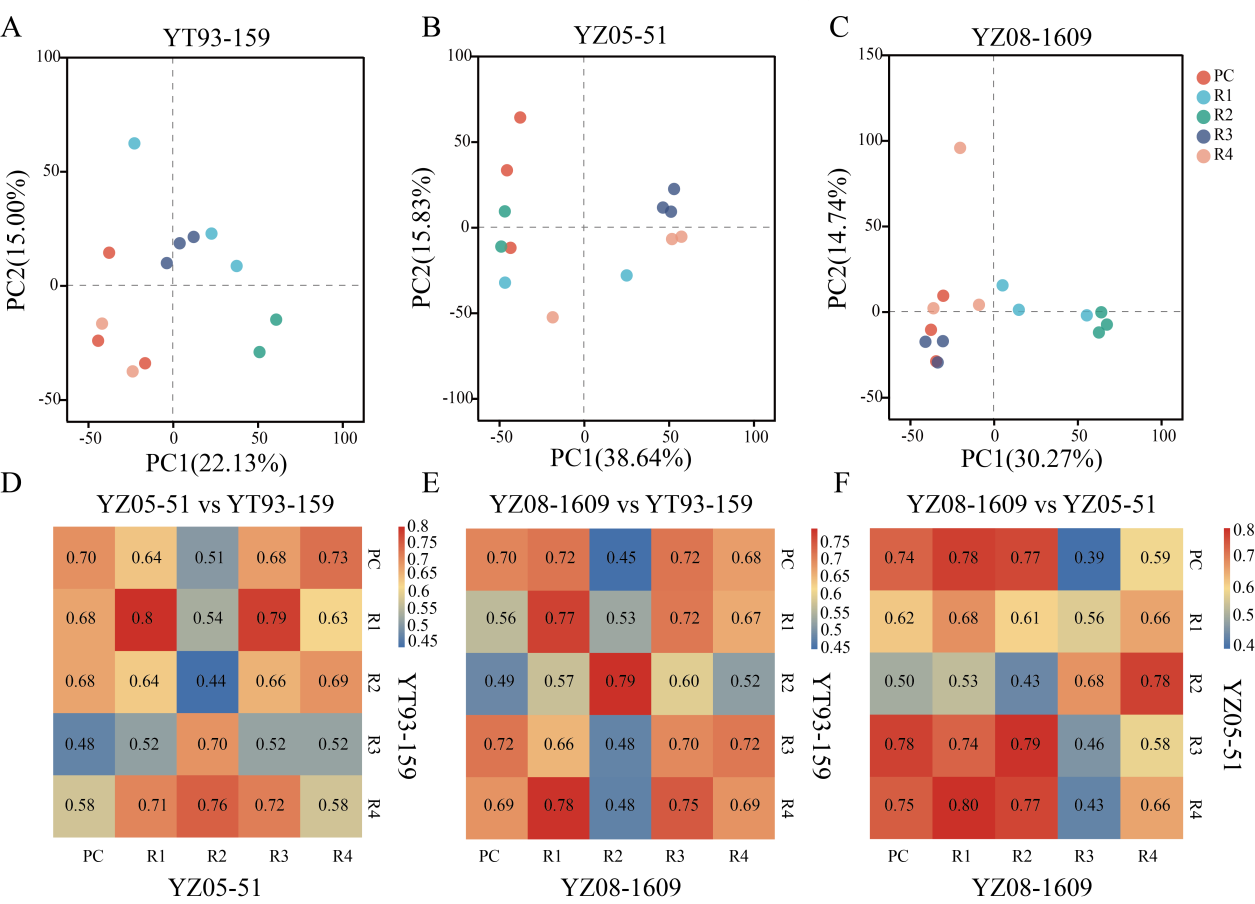


Figure S3. Principal component analysis of three sugarcane varieties respectively, and Pearson correlation coefficient analysis between each pair of varieties. (A–C) Principal component analysis (PCA) of transcriptome samples from three sugarcane varieties: YT93-159 (A), YZ05-51 (B), and YZ08-1609 (C). Each dot represents an individual biological sample. Colors indicate different planting years: red = PC (plant cane), sky blue = R1 (first ratoon year), green = R2 (second ratoon year), purple-blue = R3 (third ratoon year), and light orange = R4 (fourth ratoon year). Percentages on the axes indicate the proportion of variance explained by each principal component.

(D–F) Heatmaps showing Pearson correlation coefficients between gene expression profiles of different varieties at corresponding planting years: YZ05-51 vs YT93-159 (D), YZ08-1609 vs YT93-159 (E), and YZ08-1609 vs YZ05-51 (F). Each cell displays the correlation coefficient (r) between samples from the two varieties at the indicated planting years (PC, R1–R4). Color scale represents correlation strength: blue (low, r ≈ 0.4-0.5), yellow (moderate, r ≈ 0.6-0.7), to red (high, r ≈ 0.8), with numerical values shown in each cell. Higher correlation coefficients indicate greater similarity in gene expression patterns between varieties at the corresponding planting year.


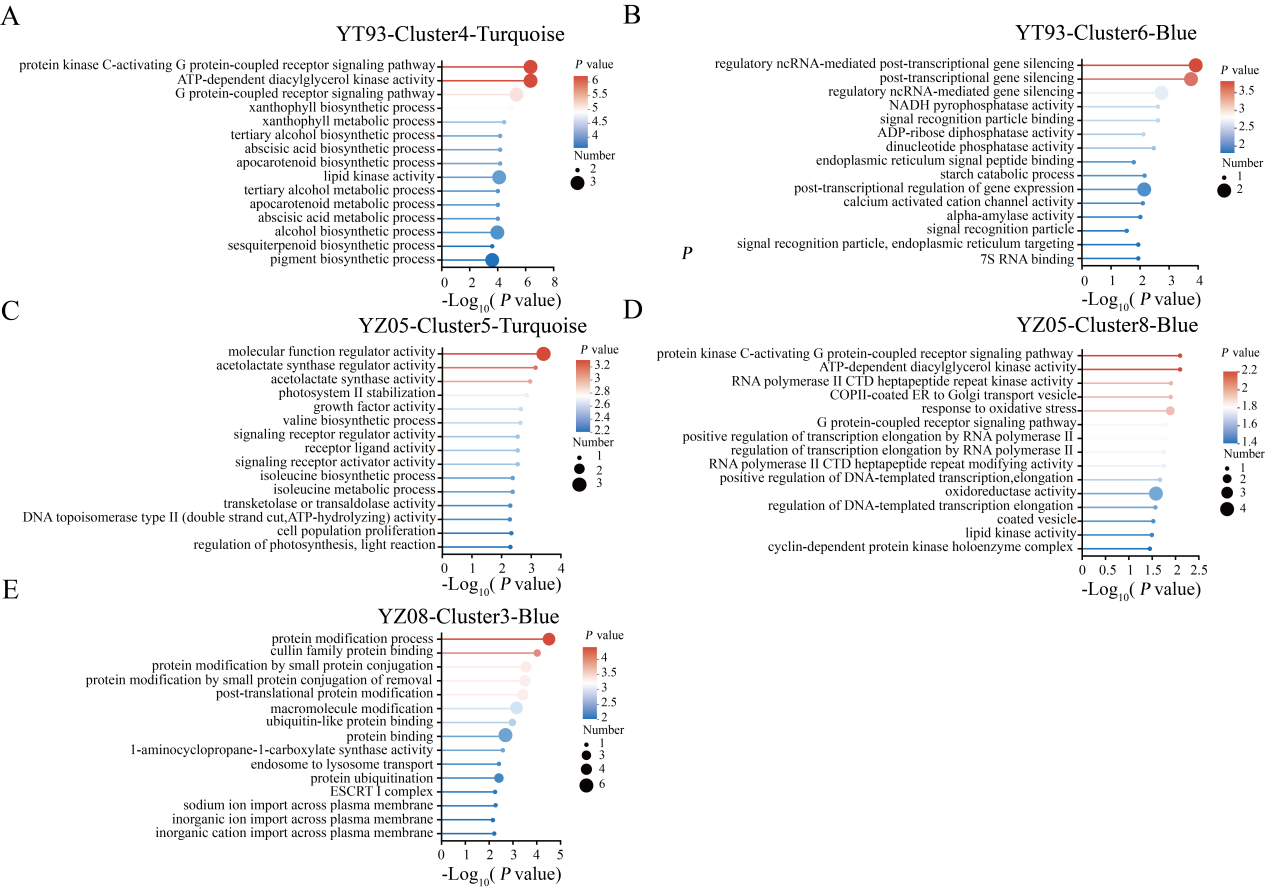
Figure S4. GO enrichment of YT93-Cluster4-Turquoise (A), YT93-Cluster6-Blue (B), YZ05-Cluster5-Turquoise (C), YZ05-Cluster8-Blue (D), and YZ08-Cluster3-Blue (E). The X-axis represents -log_10_(*P*‐value), indicating the significance of enrichment, and the Y-axis shows the enriched GO terms. Dot size represents the number of genes enriched for the corresponding term, and dot color indicates the *P*‐value (redder colors denote smaller *P* values and more significant enrichment).


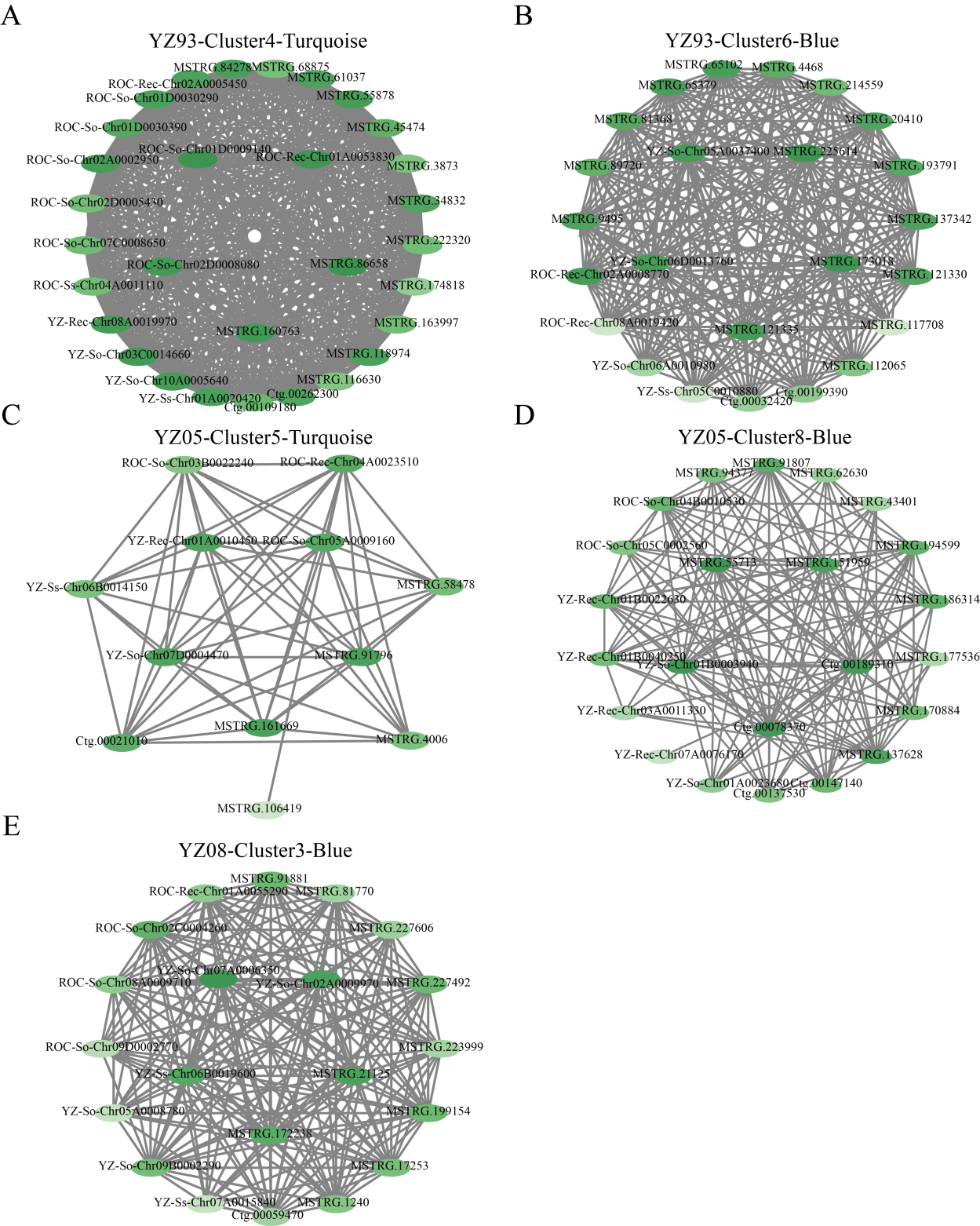


Figure S5. Gene co-expression correlation networks for YZ93-Cluster4-Turquoise (A), YZ93-Cluster6-Blue (B), YZ05-Cluster5-Turquoise (C), YZ05-Cluster8-Blue (D), and YZ08-Cluster3-Blue (E). Green circles represent gene nodes; the five larger nodes positioned centrally correspond to the top 5 hub genes ranked by module membership (kME) values. Edges between nodes indicate significant co-expression correlations.
